# CT radiomics nomogram for the preoperative prediction of severe post-hepatectomy liver failure in patients with huge (≥ 10 cm) hepatocellular carcinoma

**DOI:** 10.1186/s12957-021-02459-0

**Published:** 2021-12-12

**Authors:** Fei Xiang, Xiaoyuan Liang, Lili Yang, Xingyu Liu, Sheng Yan

**Affiliations:** 1 Department of Hepatobiliary Pancreatic Surgery, Second Affiliated Hospital, Zhejiang University School of Medicine, Hangzhou, 310003 China; 2 Department of Radiology, First Affiliated Hospital, Zhejiang University School of Medicine, Hangzhou, 310003 China

**Keywords:** Hepatocellular carcinoma, Liver failure, Radiomics, Nomogram, Decision tree

## Abstract

**Background:**

This study aimed to establish a radiomics-based nomogram for predicting severe (grade B or C) post-hepatectomy liver failure (PHLF) in patients with huge (≥ 10 cm) hepatocellular carcinoma (HCC).

**Methods:**

One hundred eighty-six patients with huge HCC (training dataset, *n* = 131 and test dataset, *n* = 55) that underwent curative hepatic resection were included in this study. The least absolute shrinkage and selection operator (LASSO) approach was applied to develop a radiomics signature for grade B or C PHLF prediction using the training dataset. A multivariable logistic regression model was used by incorporating radiomics signature and other clinical predictors to establish a radiomics nomogram. Decision tree analysis was performed to stratify the risk for severe PHLF.

**Results:**

The radiomics signature consisting of nine features predicted severe PHLF with AUCs of 0.766 and 0.745 for the training and test datasets. The radiomics nomogram was generated by integrating the radiomics signature, the extent of resection and the model for end-stage liver disease (MELD) score. The nomogram exhibited satisfactory discrimination ability, with AUCs of 0.842 and 0.863 for the training and test datasets, respectively. Based on decision tree analysis, patients were divided into three risk classes: low-risk patients with radiomics score < -0.247 and MELD score < 10 or radiomics score ≥ − 0.247 but underwent partial resections; intermediate-risk patients with radiomics score < − 0.247 but MELD score ≥10; high-risk patients with radiomics score ≥ − 0.247 and underwent extended resections.

**Conclusions:**

The radiomics nomogram could predict severe PHLF in huge HCC patients. A decision tree may be useful in surgical decision-making for huge HCC hepatectomy.

**Supplementary Information:**

The online version contains supplementary material available at 10.1186/s12957-021-02459-0.

## Introduction

The high prevalence of hepatitis B virus (HBV) infection in China is paralleled by an elevated incidence of hepatocellular carcinoma (HCC), accounting for approximately half of cases worldwide [1, 2]. Huge HCC (≥ 10 cm) is not uncommon due to a lack of early detection, often due to poor awareness. Studies have shown a relatively satisfactory overall survival in selected patients that underwent huge HCC hepatectomy [3–5]. However, patients with huge HCC often require major or extended liver resection, which puts them at high risk of post-hepatectomy liver failure (PHLF).

PHLF is a predominant cause of postoperative mortality, with reported mortality rates as high as 50% [6], and is associated with a prolonged hospital stay, compromised long-term overall survival, and increased costs in patients undergoing this surgical procedure. To prevent PHLF, a detailed assessment of liver function is a prerequisite for the appropriate selection of patients for hepatectomy. Numerous methods have been used to predict PHLF, including clinical parameters and scoring systems [7–9], dynamic quantitative liver function tests [10, 11], and remnant liver volume [12, 13]. However, the predictive outcomes are variable, and no single method alone can accurately predict PHLF. Therefore, establishing a comprehensive model based on multiple approaches may improve the predictive yield.

An emerging methodology named radiomics involves the high-throughput extraction of imaging features based on intensity, shape, texture, and higher-order features. Radiomics can potentially characterize diseases and guide clinical decision-making. Initially applied in oncological studies, it is increasingly used nowadays to study non-oncological diseases [14]. Recent studies substantiate that radiomics has improved the accuracy in diagnosing liver fibrosis and cirrhosis and could have significant value in assessing liver function [15, 16].

Accordingly, we sought to establish a CT-based radiomics signature and a nomogram by combining radiomics features and independent clinical factors for predicting severe (grade B or C) PHLF in patients with huge HCC.

## Materials and methods

### Patients

**Fig. 1:**
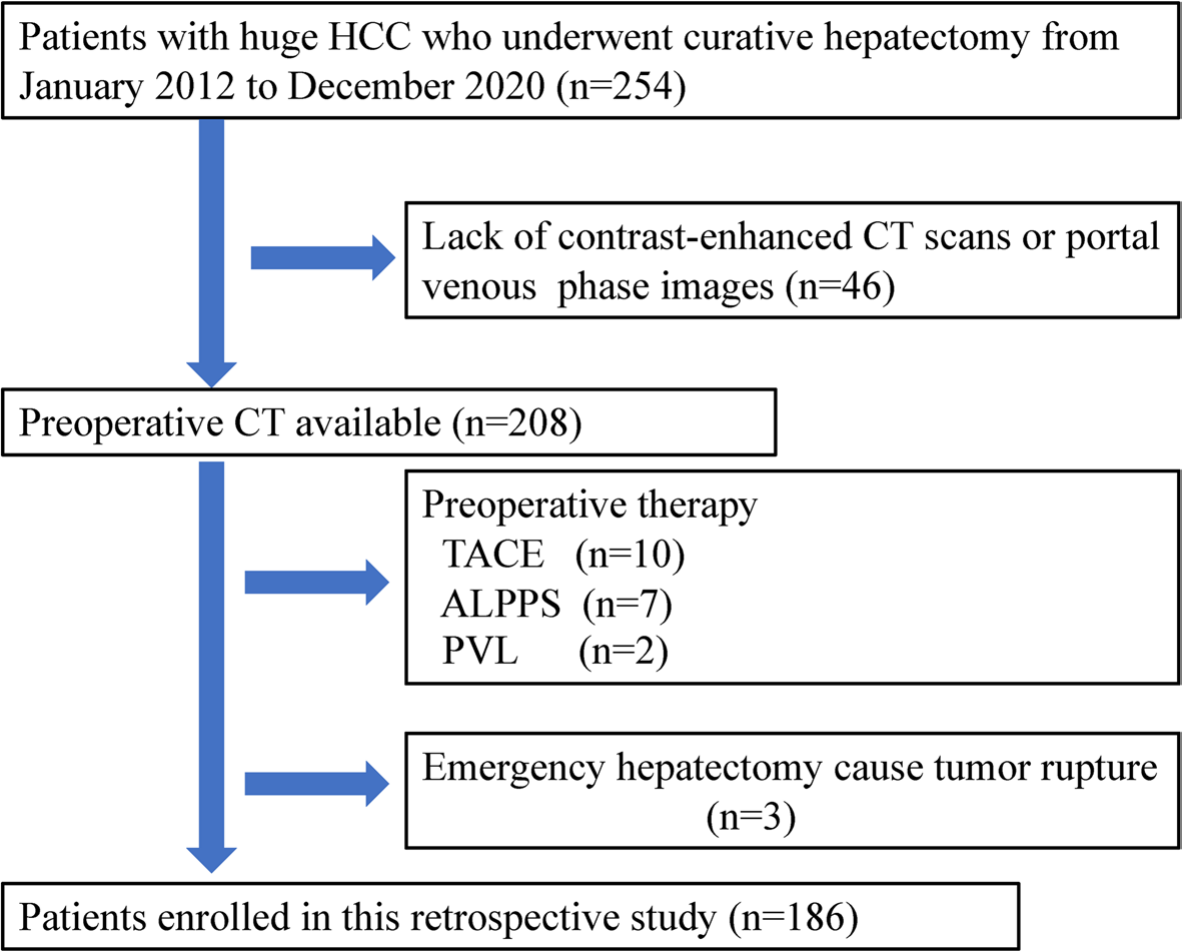
Flowchart of patients enrolled in this study. TACE, transarterial chemoembolization; ALPPS, associating liver partition and portal vein ligation for staged hepatectomy; PVL, portal vein ligation

From January 2012 to December 2020, a total of 1267 patients with HCC underwent hepatic resection in our hospital. Of these, 254 patients with huge HCC who underwent curative surgical resection were retrospectively recruited. Sixty-eight patients were excluded, and 186 patients who met the following inclusion and exclusion criteria were enrolled in this study. The inclusion criteria consisted of (1) patients who did not receive any treatment before surgery; (2) liver function was classified as Child-Pugh grade A or B; (3) Eastern Cooperative Oncology Group (ECOG) performance score 0–2; (4) patients that underwent an enhanced CT scan within 7 days before surgery; (5) patients with histologically confirmed HCC. The exclusion criteria comprised (1) no preoperative contrast-enhanced CT available or poor CT image quality; (2) patients who underwent preoperative therapy; and (3) cases of huge HCC rupture that required emergency hepatic resection. The detailed enrollment process of patients is presented in Fig. 1. Then, patients were divided into training and test datasets at a ratio of 7:3. The training dataset was used to construct the prediction model, and the test dataset was used to confirm the model’s performance. The Ethics Review Board of the Second Affiliated Hospital of Zhejiang University School of Medicine approved this study (No. 2021-0376).

### Clinical characteristics

Baseline demographic, clinical and laboratory characteristics (including liver and kidney function tests, platelet count, blood coagulation index, and serum alpha-fetoprotein level), and clinical grading scores were collected. The operative variables (including surgical methods, intraoperative blood loss, intraoperative blood transfusion, and intraoperative vascular occlusion methods) correlated with PHLF were also recorded.

### Diagnosis and definitions

PHLF was diagnosed according to the International Study Group of Liver Surgery (ISGLS) criteria [17]. The INR was set at 1.5 and the bilirubin level of more than 20 μmol/L (1.2 mg/dL). The severity of PHLF was divided into 3-classes according to the clinical management: grade A, no further clinical management necessary; grade B, requires an active therapeutic intervention without invasive approach; grade C, invasive approach. We defined grades B and C PHLF as severe PHLF, which was the primary outcome of our study since grade A PHLF does not require any additional management.

### CT scan acquisition

CT scans were performed using multi-detector CT systems (16-slice SOMATOM Perspective, SIEMENS; 16-slice SOMATOM Sensation, SIEMENS, Germany). Dynamic contrast-enhanced CT imaging was obtained following the administration of iodinated contrast material (Iohexol, GE Healthcare, USA) at 3.0 mL/s. Scanning parameters included 120 KV, 160 mAs; rotation time 0.5 s; 350 mm×350 mm field of view; matrix of 388 × 388; slice thickness, 3 mm. The arterial phase and portal phase images were obtained at 40 s and 72 s after injection of contrast medium.

### Image segmentation and radiomics features extraction

The region of interest (ROI) was drawn manually using the freely available application ITK-SNAP (version 3.6.0). ROI was delineated in the liver along the border of the whole liver parenchyma by avoiding major blood vessels, focal lesions, and artifacts on the portal phase images. Features were extracted from each segmented ROI, divided into textual and non-textural features using PyRadiomics [18], an open-source python package for medical imaging.

To obtain reproducible radiomics features, standardized computation of radiomics features was necessary [19]. In our study, the sitkBSpline interpolation was applied to resample the images with a pixel size of 1 × 1 mm. Voxel intensities were discretized using a bin-width of 25 HU. Seven hundred eighty-eight radiomics features were extracted from the liver ROI, including 18 original first-order histogram features, 14 original shape features, 68 original textural features, and 688 high-order wavelet features. The list of radiomics features is shown in Supplementary Table 1.

### Inter-observer and intra-observer agreement

To ensure reproducibility, CT images of 20 patients were randomly selected and independently resegmented by reader 1 (X.F. with 7 years of experience in liver imaging) at an interval of 2 weeks and reader 2 (YLL with 8 years of experience in liver imaging). The intra-observer reproducibility and inter-observer reliability of features extraction were assessed using intra- and inter-class correlation coefficients (ICCs). Features with ICC > 0.75 represented a good agreement and were retained.

### Feature selection and radiomics signature construction

The extracted radiomics features were normalized by the Z-score method. Radiomics features with ICCs lower than 0.75 were excluded. Univariate analyses were conducted using univariate logistic regression analysis. Features were considered to be associated with severe PHLF when the p values were less than 0.1. The least absolute shrinkage and selection operator (LASSO) algorithm was applied to identify significant features with non-zero coefficients based on the selected features. The penalty parameter (λ) was optimized through the tenfold cross-validation method. A radiomics signature was constructed by summing the selected features multiplied by their coefficients. The area under the receiver operating characteristic curve (AUC area under the ROC curve) was calculated to assess the predictive ability of the established radiomics signature.

### Development of the clinical-radiomics nomogram

To develop a comprehensive clinical-radiomics nomogram, the clinical characteristics and radiomics signature were analyzed by univariate logistic regression. Significant factors (*p* < 0.05) were used to build the multivariate logistic model. Finally, a clinical-radiomics nomogram model integrating the clinical predictors and the radiomics signature was established using the training dataset.

### Assessing the accuracy of nomogram model and comparison with conventional methods

We determined the discriminatory ability of the nomogram model by comparing the radiomics signature, albumin-bilirubin score (ALBI) score, the model for end-stage liver disease (MELD) score, and Child-Pugh score with the areas under the receiver operating characteristic curve (AUC). DeLong’s test was used to compare the nomogram model with conventional methods based on the AUC values in both datasets. To evaluate the consistency of the nomogram, we plotted a calibration curve with the Hosmer-Lemeshow goodness-of-fit test.

### Clinical use

To assist in surgical decision-making, a decision tree for safe huge HCC hepatectomy was built based on the identified risk factors. In addition, to evaluate the clinical usefulness of the nomogram model, radiomics signature, MELD, ALBI, and Child-Pugh scores, decision curve analysis (DCA) was conducted to assess the net benefits across a variety of threshold risks.

### Statistical analysis

**Fig. 2:**
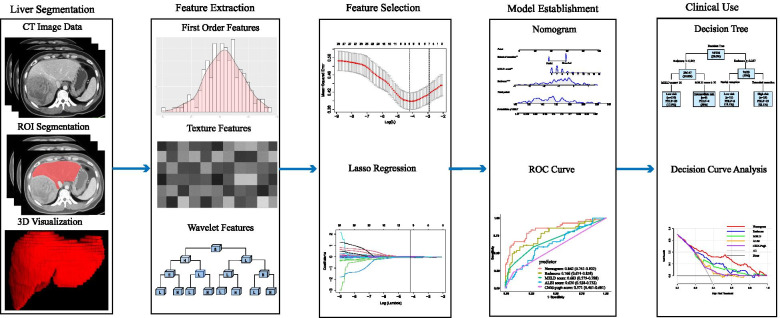
Workflow for the radiomics process. After CT images were acquired, segmentation of liver parenchyma was performed. The extracted radiomics features include intensity, shape, texture features, and wavelet features. Nine radiomics features were selected by the LASSO algorithm. A nomogram was built that incorporates radiomics signature and independent clinical predictors for individualized predicting severe PHLF. The discrimination ability of nomogram and conventional models were compared by ROC curve analysis and quantified by the AUC values. A decision tree was built to stratify the risk for severe PHLF into three classes. Clinical benefits of nomogram and conventional models were compared by decision curve analysis

The radiomics analysis workflow is shown in Fig. 2. Continuous variables and categorical variables were compared by Mann–Whitney *U* test and the chi-square test, respectively. Two-tailed values of *p* < 0.05 were statistically significant for all analyses. All analyses were conducted using R software (version 3.6.1).

## Results

### Patient demographic

A total of 186 patients (71 men, 66 women) were included in the present study. The patients were assigned to training (*n* = 131) and test datasets (*n* = 55) at a ratio of 7:3. The clinical variables did not differ significantly between the two datasets, except for HBsAg positivity (*P* = 0.044) and intraoperative blood transfusion (*P* = 0.002). The percentage of severe PHLF was 31.3% (*n* = 41) and 23.64% (*n* = 13) in the training and test datasets, respectively. The baseline characteristics are presented in Table 1.

**Table 1:** Comparison of patient demographics and clinicopathological features of the two datasets

Variable	Training dataset (*n* = 131)	Test dataset (*n* = 55)	*p* value
Sex, *n* (%)			0.363
Male	118 (90.08)	47 (85.46)	
Female	13 (9.92)	8 (14.54)	
Age, years	58.69 ± 11.13	53.25 ± 17.19	0.387
BMI, kg/m^2^	23.97 ± 3.79	23.89 ± 4.01	0.963
HBsAg positive, *n* (%)	104 (79.38)	36 (65.45)	**0.044**
Child-Pugh class, *n* (%)			0.923
A	115 (87.79)	48 (87.27)	
B	16(12.21)	7(12.72)	
AFP, median (IQR), ng/ml	38.7 (4–3849)	171.5 (9–9312)	0.717
Liver function tests			
ALB (g/L)	38.07 ± 4.84	37.55 ± 6.57	0.837
TBIL (μmol/L)	16.07 ± 8.75	18.94 ± 12.43	0.541
ALT (U/L)	48.00 ± 53.05	58.88 ± 47.64	0.641
AST (U/L)	81.23 ± 63.99	75.75 ± 66.02	0.853
GGT(U/L)	130.62 ± 109.54	167.13 ± 166.12	0.549
Cr (μmol/L)	50.46 ± 11.81	55.63 ± 12.83	0.358
PLT(10^9^/L)	202.92 ± 83.81	198.50 ± 63.50	0.900
PT (s)	13.45 ± 1.32	12.59 ± 1.26	0.155
INR	1.04 ± 0.12	1.02 ± 0.96	0.631
Tumor size, mm	125.54 ± 25.78	127.13 ± 52.94	0.930
Cirrhosis, *n* (%)	76 (58.02)	29 (52.72)	0.507
Extent of resection			0.515
Extended (≥ 4 segments)	84 (64.12)	38 (69.09)	
Partial (< 4 segments)	47 (35.88)	17 (30.91)	
Conventional predictive models			
Child-Pugh score^a^	5 (5–8)	5 (5–8)	0.463
MELD score^a^	7 (6–15)	7 (6–13)	0.568
ALBI score^b^	− 2.47 (− 0.51~− 3.42)	− 2.55 (− 1.09~− 3.32)	1.000
Intraoperative blood loss, ml ^b^	400 (200–800)	500 (300–800)	0.199
Intraoperative blood transfusion, *n* (%)			
Yes	87 (66.4%)	23 (41.8%)	**0.002**
No	44 (33.6%)	32 (58.2%)	
Pringle maneuver, *n* (%)			0.872
Yes	65 (49.6%)	28 (50.9%)	
No	66 (50.4%)	27 (49.1%)	
PHLF (B/C), *n* (%)			0.293
Yes	41 (31.30)	13 (23.64)	
No	90 (68.70)	42 (76.36)	
Postoperative mortality, *n* (%)	5 (3.8%)	3 (5.4%)	0.696

*BMI* body mass index, *HBsAg* hepatitis B surface antigen, *AFP* alpha fetoprotein, *SD* standard deviation, *ALB* albumin, *TBIL* total bilirubin, *ALT* alanine aminotransferase, *AST* aspartate transaminase, *GGT* γ-glutamyl transpeptidase, *PLT* platelets, *PT* prothrombin time, *INR* international normalized ratio, *MELD* model for end-stage liver disease, *ALBI* albumin to bilirubin ration index, *PHLF* posthepatectomy liver failure

^a^Median (range)

^b^Median (IQR)

### Radiomics signature construction

**Fig. 3:**
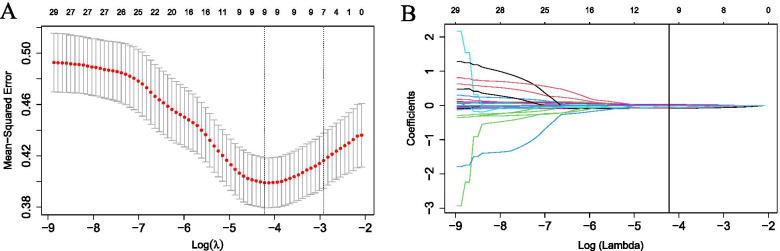
The LASSO algorithm was used to select predictive radiomics features. **A** Tuning parameter (λ) in the LASSO model was selected by ten-fold cross-validation. The optimal λ value of 0.015 with log(λ) of − 4.269 was chosen (at the minimum criteria). **B** Coefficients of 30 features were shrunk with the penalty term increases. Nine features with nonzero coefficients were obtained with the optimal λ

Of the 788 extracted radiomics features, 165 features were eliminated due to an ICC lower than 0.75. Subsequently, univariate logistic regression was used to select PHLF-associated features. Thirty features remained and were subjected to LASSO regression to screen for critical features and construct the radiomics signature. Finally, nine features with non-zero coefficients were screened by the LASSO approach using the training dataset (Fig. 3A, B). Among the nine features, two features were original shape features, and the remaining were wavelet features. The radiomics signature was constructed using the nine features, and the radiomics score was computed as follows:

- Radscore = − 0.93044761 + 0.20910827 * original_shape_Maximum2DDiameterSlice + 0.04625660 * original_shape_SurfaceVolumeRatio − 0.08693156 * HHH_glszm_ZoneVariance − 0.44200827 *HHL_firstorder_Median − 0.42800711*HHL_gldm_DependenpendenceNonUniformityNormalized − 0.04493315 *HLH_firstorder_Maximum − 0.35475442*HLH_glcm_ClusterProminence + 0.01233872 * LHH_glszm_LowGray − LevelZoneEmphasis − 0.36996067*LLH_glszm_GrayLevelNonUniformity

In patients with PHLF, the Radscore (median [range]) was significantly higher than non-PHLF patients in the training dataset (− 0.290 − 2.443∼1.462] vs. − 1.067 [− 3.686∼0.404], respectively, *P* < 0.001). The same trend was observed in the test dataset (− 0.536 [− 1.375∼1.461] vs. − 0.930 [− 3.875∼1.138], respectively, *P* = 0.007). The distributions of Radscore for each patient in the training and test datasets are shown in Supplementary Fig. 1.

### Development of the clinical-radiomics nomogram and comparison with conventional models

**Fig. 4:**
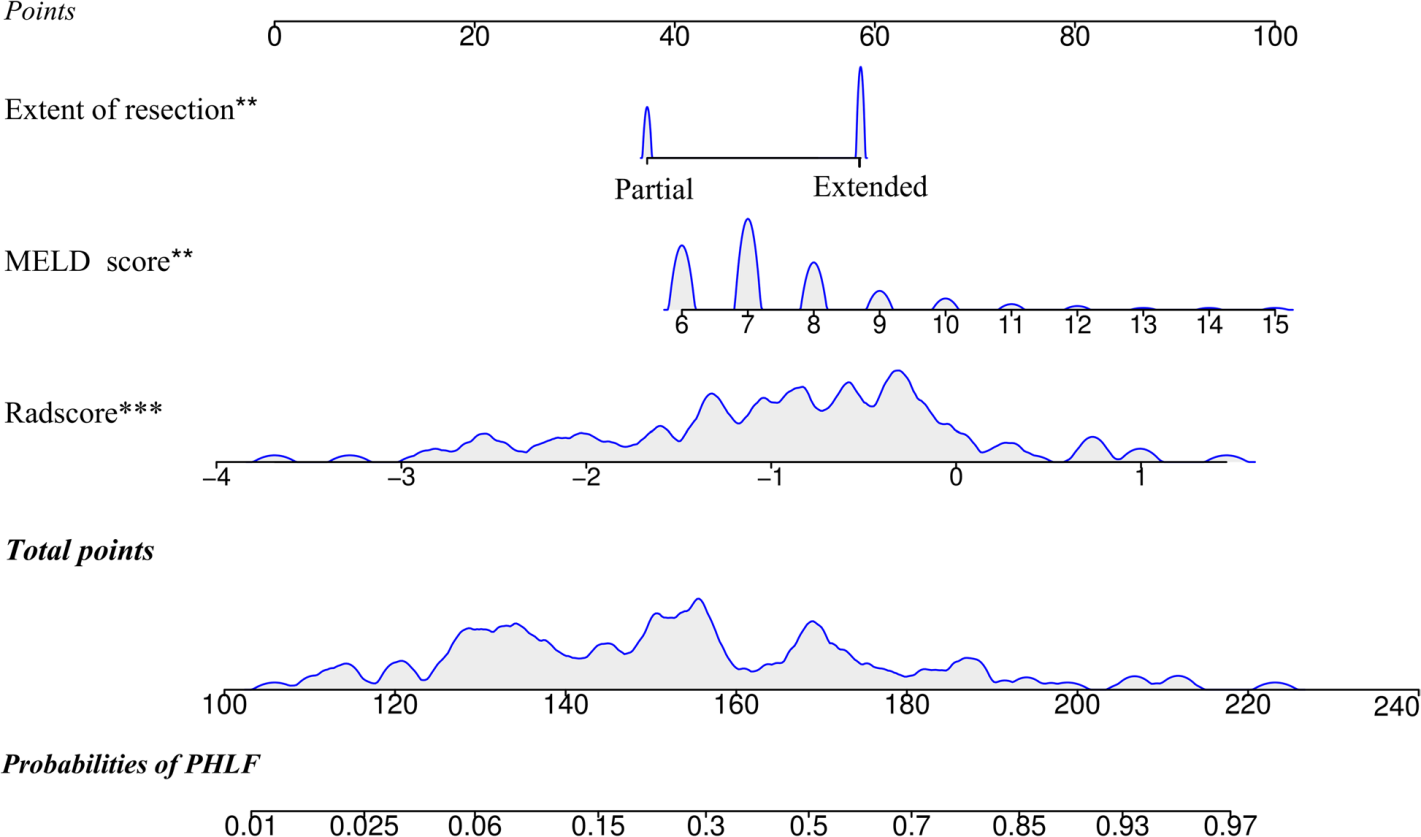
The radiomics nomogram was developed by incorporating the Radscore, the MELD score, and the extent of resection

**Fig. 5:**
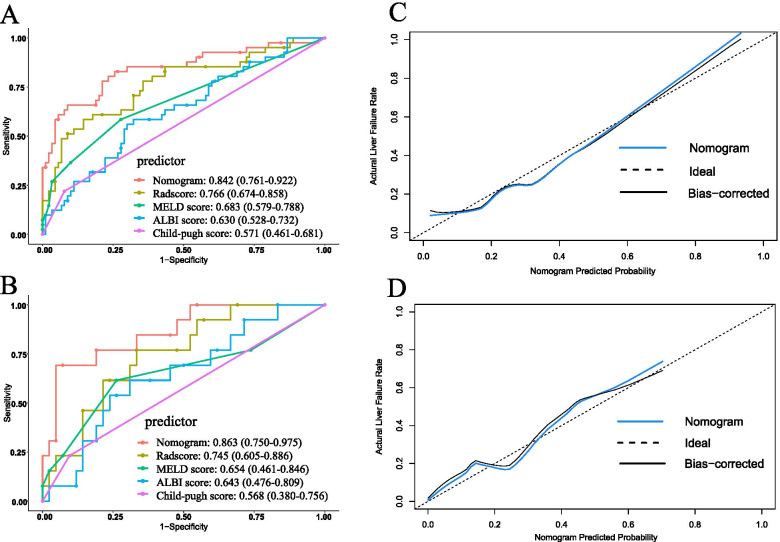
Assessing the accuracy of the nomogram model and comparison with conventional methods. The nomogram showed a significantly higher discrimination power than Radscore, MELD score, ALBI score, and Child-Pugh score for predicting severe PHLF in the training (**A**) and test (**B**) datasets. The calibration curves demonstrated good agreement between the radiomics nomogram predicted and actual observation in the training (**C**) and test (**D**) datasets

Univariate and multivariate logistic regression analysis found that Radscore, MELD score, and the extent of resection were significant predictive factors of severe PHLF (Table 2). An individualized nomogram model was developed using these significant independent risk factors (Fig. 4). The nomogram showed good discrimination ability, with a mean AUC of 0.842 (95% confidence interval (CI): 0.761–0.922) and 0.863 (95% CI 0.750–0.975) in the training (Fig. 5A) and test datasets (Fig. 5B). In the training dataset, the nomogram model yielded a significantly higher AUC than the Child-Pugh score (*P* < 0.001), MELD score (*P* = 0.001), and ALBI score (*P* < 0.001). Similar results were found with the test dataset (nomogram vs. Child-Pugh score, *P* < 0.001; nomogram vs. MELD score, *P* = 0.002; nomogram vs. ALBI score; *P* = 0.02). The calibration curve showed good agreement between the predicted and actual observations in the training and test datasets (Fig. 5C, D). Moreover, the *p* value of the Hosmer-Lemeshow test was 0.397 and 0.285 in the training and test datasets, suggesting a good fit between the nomogram and actual observations.

**Table 2:** Univariable and multivariable logistic regression analyses of risk factors for severe PHLF in the training dataset

Variables	Univariate analysis			Multivariate analysis		
	OR	95%CI	*P* value	OR	95% CI	*P* value
Age	1.012	0.962–1.066	0.639			
Sex, male vs female	2.470	0.456–13.379	0.294			
BMI (≥ 25 vs < 25)	0.628	0.182–2.160	0.460			
HBV infection	1.703	0.419–6.922	0.457			
TBIL	0.949	0.871–1.034	0.232			
ALB	0.965	0.844–1.103	0.603			
PT	1.512	0.809–2.829	0.195			
INR (per 0.1 increase)	0.707	0.327–1.529	0.378			
PLT	0.999	0.993–1.006	0.820			
Tumor size	0.996	0.976–1.018	0.741			
Cirrhosis	0.660	0.177–2.465	0.537			
Extent of resection (extended vs partial)	4.903	1.381–17.405	**0.014**	4.483	1.591–12.633	**0.005**
Blood loss (≥ 800 vs < 800 ml)	0.250	0.054–1.161	0.077			
Blood transfusion	2.609	0.517–13.164	0.245			
Pringle maneuver	1.217	0.394–3.763	0.733			
Child-Pugh score	2.803	0.239–32.839	0.412			
MELD score	1.891	1.093–3.271	**0.023**	1.589	1.189–2.124	**0.002**
ALBI score	0.955	0.355–2.725	0.931			
Radscore (per 0.1 increase)	1.144	1.068–1.224	**< 0.001**	1.139	1.066–1.216	**< 0.001**

### Clinical use

**Fig. 6:**
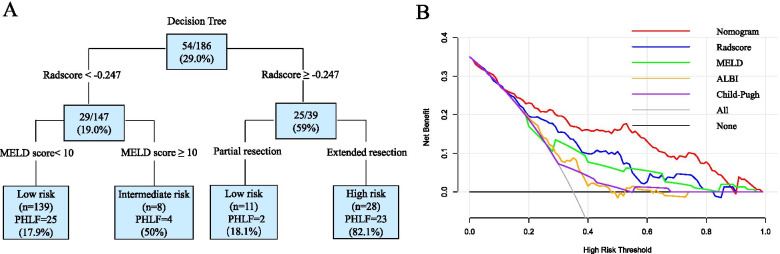
Clinical use. **A** The decision tree stratified the risk for severe PHLF into three classes. **B** DCA showed that the nomogram had wider threshold probabilities and yielded more net benefit than conventional models

Decision tree analysis stratified the risk for severe PHLF based on the Radscore, MELD score, and the extent of resection into three classes (Fig. 6A). For low-risk patients with radiomics score < − 0.247 and MELD score < 10 or radiomics score ≥ − 0.247 but underwent partial resections, the probability of severe PHLF was 18%. For intermediate-risk patients with radiomics score <− 0.247 but MELD score ≥ 10, the likelihood of severe PHLF was 50%. Finally, for high-risk patients with radiomics score ≥− 0.247 that underwent extended resections, the probability of severe PHLF was 82%. Importantly, DCA (Fig. 6B) showed that our nomogram has a high potential for clinical application with wider threshold probabilities than conventional models.

## Discussion

The present study established a radiomics signature for the individual preoperative prediction of severe PHLF for patients that undergo huge HCC hepatectomy. We then developed a clinical-radiomics nomogram comprising the radiomics signature and clinical predictors. The nomogram model integrated three predictive variables that could reflect the preoperative clinical essentials, which yielded good predictive ability for severe PHLF. Based on radiomics score, MELD score, and the extent of resection, a decision tree was built, and the whole series was split into three risk groups.

In recent years, improved hepatic resection techniques and expanded surgical indications have acted as a prelude to an increase in extensive liver resection, leading to a higher risk of PHLF. Single-center studies have reported that the PHLF risk ranged between 25.8% and 35.3%, while severe PHLF ranged between 11.3% and 28% [20–23]. Due to large tumor diameters and major vascular invasion, approximately 62% to 80% of patients with huge HCC undergo major or extensive liver resection leading to morbidity and mortality rates in the range of 10.9–43.6% and 4.2–18.1% [24–27]. Therefore, establishing an individualized prediction model for PHLF in patients with huge HCC is critical.

Radiomics is a high-throughput data mining method that involves extracting features from medical images and is extensively used in oncological studies. Radiomics quantitatively assesses tumor heterogeneity by reflecting the distribution of gray level values and spatial arrangement of the pixels. Besides, in recent years, it has gradually been applied for the study of non-oncological diseases. In chronic liver diseases, studies have demonstrated the potential benefits of radiomics in assessing liver parenchyma heterogeneity, reflecting architectural disturbance to predict liver function [28]. For instance, radiomics of shear wave elastography, MRI, and CT have been used to assess liver fibrosis quantitatively and have shown good diagnostic accuracy, irrespective of the imaging modality [15, 16, 29]. Furthermore, radiomic features have been used to predict the occurrence of PHLF. In this regard, a study by Pak [30] reported that the liver parenchyma in patients with PHLF exhibited a more heterogeneous appearance, with wide variations in pixel intensities. In contrast, a more homogenous liver appearance was documented in normal patients. Importantly, with the help of machine learning, significant features can be selected and established as radiomics signatures. In a study by Cai et al. [31] where the radiomics score was calculated using CT-based higher-order wavelet features, the AUCs for the prediction of PHLF were 0.82 and 0.76 in the training and validation groups, respectively. Besides, Zhu et al. [32] reported an MRI-based radiomics model which combined first order and texture features associated with PHLF, resulting in an accuracy of 80.9% during validation. Similarly [33], a liver failure model developed by Chen et al. incorporated PLT count, tumor size, and radiomics features from Gd-EOB-DTPA-enhanced MRI images and yielded better performance than the conventional clinical model. We reviewed these studies and compared the outcomes in Supplementary Table 2. Unlike these studies, grade A PHLF was not included in our study since patients with grade A PHLF tended to be asymptomatic and did not require specific treatments. Based on our experience, we are convinced that predicting symptomatic grade B or C PHLF is more valuable to guide surgeons during the decision-making process.

Herein, various prediction models from the literature were compared to our model. Indeed, conventional scoring systems, in combination with laboratory biochemical parameters, have valuable diagnostic value. However, conventional scoring systems only provide a rough estimate of liver function. Moreover, a single scoring system often does not fully capture the liver function status. To accurately predict PHLF, integrated models that consider patient, liver, and surgery-related risk factors are needed [34]. To this end, we established a combined nomogram model that integrated radiomics score and other clinical factors. In our nomogram model, three independent indicators, including radiomics, MELD, and extent of hepatectomy, were incorporated during multivariate logistic regression. The radiomics score was calculated using wavelet and liver shape features. The wavelet features exhibited higher weights in the radiomics score, and evidence has shown that wavelet transformation can further reflect the spatial heterogeneity across multiple dimensions [35]. Even though the MELD score has been criticized for several reasons, evidence shows that it presents good predictive accuracy for severe liver diseases [8]. Besides, numerous studies demonstrate that the MELD score is a significant factor in predicting PHLF and can be integrated with other factors to enhance the prediction accuracy [36, 37]. It has been established that extended hepatectomy is a risk factor for PHLF [22]. Moreover, the incidence of PHLF is reported to increase with the number of segments resected [38].

In our study, a decision tree was built to further assist clinical decision-making by using these factors as determinants for risk stratification. As the root node of the decision tree, the radiomics score was the most important factor associated with severe PHLF, according to the results of multivariate regression analysis. The cutoff of the radiomics score was − 0.247. Patients that underwent extended resections with a radiomics score greater than − 0.247 were classified as high risk and experienced an 82.1% risk of severe PHLF. The above findings suggest that the decision to perform surgery should be made with caution, and local treatment approaches should be considered. For patients with an intermediate risk, with a radiomics score < − 0.247 but MELD score ≥ 10, additional clinical and diagnostic information is required to determine whether hepatectomy will confer additional benefit. Clinical decision-making is straightforward for low-risk patients if there is evidence that the patient can benefit from surgery. We advocate that the decision tree model is easy to understand and manipulate by generating a set of “if-then” rules. Most importantly, the classification results can simplify the decision-making process.

One major limitation of this study is the retrospective nature that may be a source of selection bias. Another limitation is the lack of external validation using data from other hospitals. Therefore, further prospective multi-institutional studies should be conducted to assess the value of the radiomics nomogram in predicting severe PHLF and increase the robustness of our findings.

## Conclusion

The proposed clinical-radiomics nomogram, which integrates a radiomics signature and clinical predictors, yielded satisfactory discrimination and calibration power in predicting severe PHLF. The radiomics nomogram combined with the decision tree potentially provides alternative clinical prediction and decision-making methods for hepatectomy in patients with huge HCC. We hypothesize that this radiomics nomogram and decision tree play an important complementary role in predicting severe PHLF in patients with huge HCC after hepatectomy and improve the patient-selection criteria.

## Supplementary Information

**Additional file 1 **: **Supplemental Table 1**. Detailed information of extracted radiomics features. **Supplemental Figure 1**. Boxplot diagrams show that the value of the Rad-score is significantly higher in patients with severe PHLF in the training dataset (A) (*p* < 0.001) and the test dataset (B) (*p* = 0.007). **Supplemental Table 2**. Advancements and details in prediction of PHLF in each study through radiomics.

## Data Availability

The raw data of this paper are available upon reasonable request to the corresponding author.

## Abbreviations

PHLF: Post-hepatectomy liver failure
HCC: Hepatocellular carcinoma
AUC: The areas under the receiver operating characteristic curve
MELD: The extent of resection and model for end-stage liver disease
HBV: Hepatitis B virus
ECOG: Eastern Cooperative Oncology Group
ISGLS: International Study Group of Liver Surgery
ROI: Region of interest
ICC: Intra- and inter-class correlation coefficients
LASSO: Least absolute shrinkage and selection operator
ALBI: Albumin-bilirubin
DCA: Decision curve analysis
Radscore: Radiomics score
