# Supplementary material for: CT radiomics nomogram for the preoperative prediction of severe post-hepatectomy liver failure in patients with huge (≥ 10 cm) hepatocellular carcinoma
Source: World J Surg Oncol. 2021 Dec 12;19:344. doi: 10.1186/s12957-021-02459-0 (PMC8667454; doi:10.1186/s12957-021-02459-0)
Supplement: Supplementary file 1 — Additional file 1 : Supplemental Table 1. Detailed information of extracted radiomics features. Supplemental Figure 1. Boxplot diagrams show that the value of the Rad-score is significantly higher in patients with severe PHLF in the training dataset (A) (p < 0.001) and the test dataset (B) (p = 0.007). Supplemental Table 2. Advancements and details in prediction of PHLF in each study through radiomics. [file 12957_2021_2459_MOESM1_ESM.docx]

**Supplementary material**

Supplemental Table 1. Detailed information of extracted radiomics features.

| **feature type** | **feature name** |
| --- | --- |
| First-order features (n=18) | 1. 10Percentile 2. 90Percentile 3. Energy 4. Entropy 5. InterquartileRange 6. Kurtosis 7. Maximum 8. MeanAbsoluteDeviation 9. Mean 10. Median 11. Minimum 12. Range 13. RobustMeanAbsoluteDeviation 14. RootMeanSquared 15. Skewness 16. TotalEnergy 17. Uniformity   18. Variance |
| Shape features(n=14) | 1. Elongation 2. Flatness 3. LeastAxisLength 4. MajorAxisLength 5. Maximum2DDiameterColumn 6. Maximum2DDiameterRow 7. Maximum2DDiameterSlice 8. Maximum3DDiameter 9. MeshVolume 10. MinorAxisLength 11. Sphericity 12. SurfaceArea 13. SurfaceVolumeRatio 14. VoxelVolume |
| Gray Level Co-occurrence Matrix (GLCM) features (n=22) | 1. Autocorrelation 2. JointAverage 3. ClusterProminence 4. ClusterShade 5. ClusterTendency 6. Contrast 7. Correlation 8. DifferenceAverage 9. DifferenceEntropy 10. DifferenceVariance 11. JointEnergy 12. JointEntropy 13. Imc1 14. Imc2 15. Idm 16. Idmn 17. Id 18. Idn 19. InverseVariance 20. MaximumProbability 21. SumEntropy 22. SumSquares |
| Gray Level Size Zone Matrix (GLSZM) features (n=16) | 1. GrayLevelNonUniformity 2. GrayLevelNonUniformityNormalized 3. GrayLevelVariance 4. HighGrayLevelZoneEmphasis 5. LargeAreaEmphasis 6. LargeAreaHighGrayLevelEmphasis 7. LargeAreaLowGrayLevelEmphasis 8. LowGrayLevelZoneEmphasis 9. SizeZoneNonUniformity 10. SizeZoneNonUniformityNormalized 11. SmallAreaEmphasis 12. SmallAreaHighGrayLevelEmphasis 13. SmallAreaLowGrayLevelEmphasis 14. ZoneEntropy 15. ZonePercentage 16. ZoneVariance |
| Gray Level Run Length Matrix (GLRLM) features (n=16) | 1. GrayLevelNonUniformity 2. GrayLevelNonUniformityNormalized 3. GrayLevelVariance 4. HighGrayLevelRunEmphasis 5. LongRunEmphasis 6. LongRunHighGrayLevelEmphasis 7. LongRunLowGrayLevelEmphasis 8. LowGrayLevelRunEmphasis 9. RunEntropy 10. RunLengthNonUniformity 11. RunLengthNonUniformityNormalized 12. RunPercentage 13. RunVariance 14. ShortRunEmphasis 15. ShortRunHighGrayLevelEmphasis 16. ShortRunLowGrayLevelEmphasis |
| Gray Level Dependence Matrix (GLDM) Features  (n=14) | 1. DependenceEntropy 2. DependenceNonUniformity 3. DependenceNonUniformityNormalized 4. DependenceVariance 5. GrayLevelNonUniformity 6. GrayLevelVariance 7. HighGrayLevelEmphasis 8. LargeDependenceEmphasis 9. LargeDependenceHighGrayLevelEmphasis 10. LargeDependenceLowGrayLevelEmphasis 11. LowGrayLevelEmphasis 12. SmallDependenceEmphasis 13. SmallDependenceHighGrayLevelEmphasis 14. SmallDependenceLowGrayLevelEmphasis |
| Wavelet features (n=688) | (1) LLL; (2) LLH; (3) LHL; (4) LHH;  (5) HLL; (6) HLH; (7) HHL; (8) HHH |
|  | For each decomposition, First-order features, GLCM，GLSZM，GLRLM，GLDM were extracted on the above 8 filtered images. Therefore, the total number of wavelet features could be calculated as (18+22+16+16+14) ×8= 688. |

A total of 788 radiomics features were extracted for each case. The feature pool comprised 18 original first-order histogram features, 14 original shape features, 68 original textural features, including 22 gray-level cooccurrence matrix (GLCM) features, 14 gray-level dependence matrix (GLDM) features, 16 gray-level run-length matrix (GLRLM) features, 16 gray-level size zone matrix (GLSZM) and 688 high-order wavelet features. The total number of features was calculated as follows: 18+14+68+688=788.

Supplemental Figure 1: Boxplot diagrams show that the value of the Rad-score is significantly higher in patients with severe PHLF in the training dataset (A) (p < 0.001) and the test dataset (B) (p = 0.007)


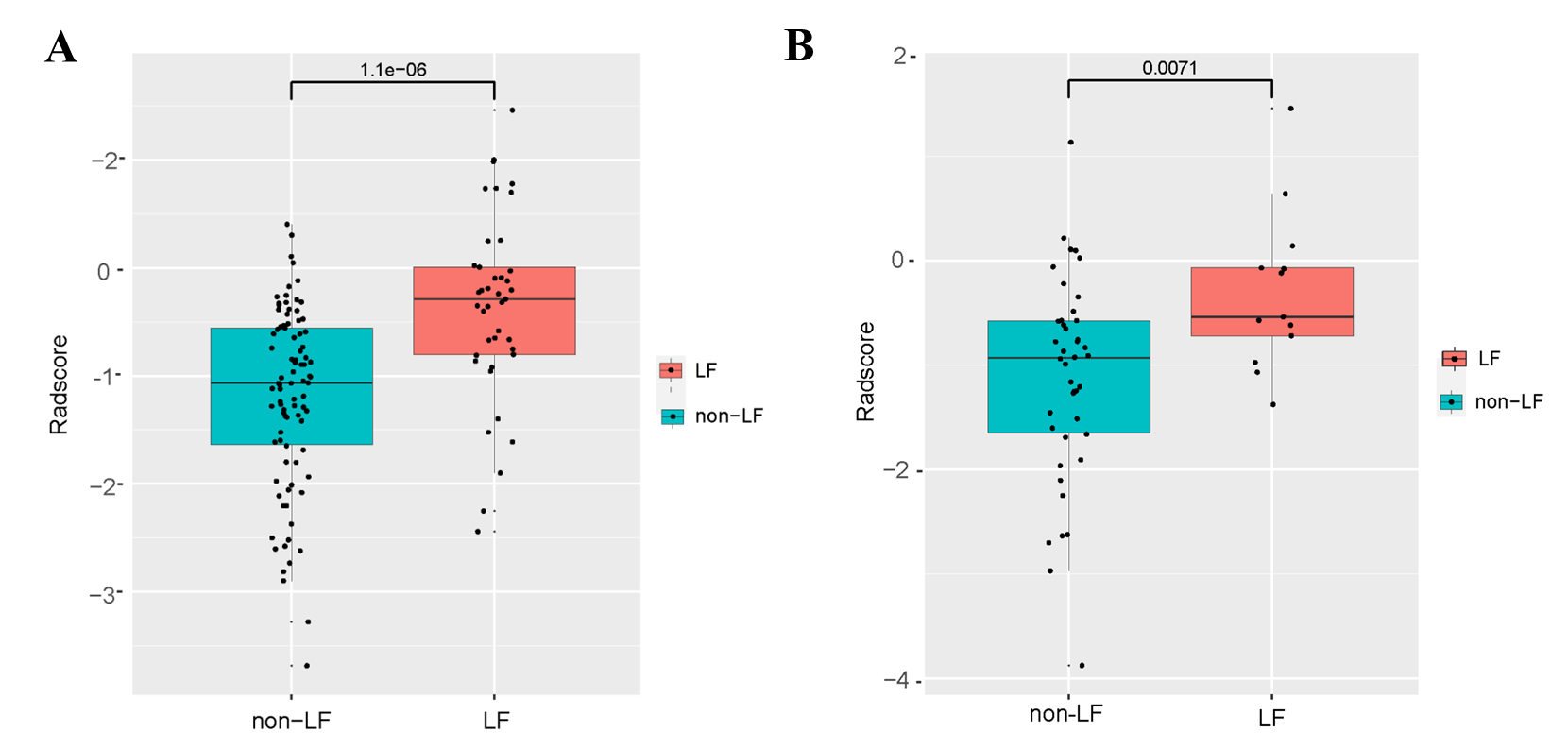


**Note:** LF, liver failure

Supplemental Table 2: Advancements and details in prediction of PHLF in each study through radiomics.

| Author | Modeling Algorithm | Imaging | Number of extracted features | Feature Signature | Independent clinical risk factors | AUC (test cohort) |
| --- | --- | --- | --- | --- | --- | --- |
| Pak [30] | without | CT | 255 | 2 radiomics features (fractal dimension 1_4,  angle co-occurrence matrices1_10) | Preoperative bilirubin,  RLV | without |
| Cai [31] | LASSO regression | CT | 713 | 7 radiomics features  (1 original shape features, and 6 wavelet features) | MELD score,  ECOG performance  score | 0.576–0.948 |
| Zhu [32] | LASSO regression | Gd-EOB-DTPA-Enhanced MRI | 61 | 5 radiomics features  (3 first order histogram features, 2 GLCM features) | ICG-R15 | 0.713-0.906 |
| Chen [33] | RFE | Gd-EOB-DTPA-Enhanced MRI | 1044 | 24 radiomics features  (3 first order histogram features, 4 GLRLM features, and 17 GLCM features) | PLT count,  Tumor size | 0.792–0.845 |
| Our study | LASSO regression | CT | 788 | 9 radiomics features  (2 original shape features, and 7 wavelet features) | The extent of resection,  MELD score | 0.674–0.858 |

**Note**: CT, computed tomography; MRI, magnetic resonance imaging; Gd-EOB-DTPA, gadolinium-ethoxybenzyl-diethylenetriamine; AUC, area under the curve; LASSO, The least absolute shrinkage and selection operator algorithm; RFE, recursive feature elimination; RLV, remnant liver volume; MELD, model for end-stage liver disease; ECOG, Eastern Cooperative Oncology Group; ICG, indocyanine green; PLT, platelets.
